# Nanostructure Sn/C Composite High-Performance Negative Electrode for Lithium Storage

**DOI:** 10.3390/molecules27134083

**Published:** 2022-06-24

**Authors:** Jaffer Saddique, Honglie Shen, Jiawei Ge, Xiaomin Huo, Nasir Rahman, Ahmad Aziz Al Ahmadi, Muhammad Mushtaq

**Affiliations:** 1 Jiangsu Key Laboratory of Materials and Technology for Energy Conversion, College of Materials Science and Technology, Nanjing University of Aeronautics and Astronautics, Nanjing 210016, China; jaffer@nuaa.edu.cn (J.S.); 18551755923@sina.cn (J.G.); huoxm0928@163.com (X.H.); 2 Dalian Product Quality Inspection and Testing Institute Co., Ltd., Dalian 116021, China; 3 Department of Physics, University of Lakki Marwat, Lakki Marwat 28420, Pakistan; nasir@ulm.edu.pk; 4 Department of Electrical Engineering, College of Engineering, Taif University, Taif 21944, Saudi Arabia; aziz@tu.edu.sa; 5 Key Laboratory of Advanced Functional Materials of Education Ministry of China, College of Materials Science and Engineering, Beijing University of Technology, Beijing 100124, China; mushtaqphy009@yahoo.com

**Keywords:** Sn-based anode material, synthesis, structural characterization, lithium-ion battery (LIB), electrochemical performance

## Abstract

Tin-based nanocomposite materials embedded in carbon frameworks can be used as effective negative electrode materials for lithium-ion batteries (LIBs), owing to their high theoretical capacities with stable cycle performance. In this work, a low-cost and productive facile hydrothermal method was employed for the preparation of a Sn/C nanocomposite, in which Sn particles (sized in nanometers) were uniformly dispersed in the conductive carbon matrix. The as-prepared Sn/C nanocomposite displayed a considerable reversible capacity of 877 mAhg^−1^ at 0.1 Ag^−1^ with a high first cycle charge/discharge coulombic efficiency of about 77%, and showed 668 mAh/g even at a relatively high current density of 0.5 Ag^−1^ after 100 cycles. Furthermore, excellent rate capability performance was achieved for 806, 697, 630, 516, and 354 mAhg^−1^ at current densities 0.1, 0.25, 0.5, 0.75, and 1 Ag^−1^, respectively. This outstanding and significantly improved electrochemical performance is attributed to the good distribution of Sn nanoparticles in the carbon framework, which helped to produce Sn/C nanocomposite next-generation negative electrodes for lithium-ion storage.

## 1. Introduction

In the last few decades, energy demands have rapidly increased due to the growing demand for portable electronic devices, such as laptops, mobile phones, cameras, etc., which are commonly used [1,2,3,4]. Moreover, the introduction of battery vehicles, including plug-in hybrid electric vehicles (PHEVs) and hybrid electric vehicles (HEVs), have contributed to the increasing demand for energy. Lithium-ion batteries (LIBs) were commercialized for the first time in 1991 by Sony Corporation, and now play a vital role in meeting the global energy demand [5,6,7,8,9]. Generally, LIBs offer a higher energy density, longer lifespan, and lighter design, and are more environmentally friendly compared with other secondary metal-ion batteries [10,11]. However, there are still improvements required to increase the energy density of LIBs, because the current commercial graphite (372 mAhg^−1^) is not sufficient to meet the increasing requirements for energy storage systems [12,13,14]. Therefore, it is necessary to design and develop new kinds of anode materials with a higher specific capacity and stable performance in order to meet the industrialization requirements at the commercial level. Metallic tin (Sn) is nontoxic and inexpensive, and has a highly specific theoretical capacity (994 mAhg^−1^); it is considered one of the alternative anode materials in LIBs [15,16]. One drawback of using Sn in LIBs is that during alloying/dealloying with lithium (Li), it undergoes massive volume expansion with low ionic conductivity, and a pulverization phenomenon occurs, in which it loses contact with the active material and current collector. This results in low cycle performance, a quickly fading capacity, and low cycle life of the battery [17]. Different strategies have been employed to overcome these problems, such as fabricating nanostructured Sn-based materials with reduced internal strain, and creating short pathways for lithium diffusion, such as Sn-based nanospheres [18], nanowires [19], and nanotubes [20]. In addition to these active and inactive materials, approaches have been devised to form a metal–Sn alloy and fabricate stable structures such as Co-Sn, Cu-Sn, Ni-Sn, etc. [20,21,22] Moreover, there has been significant work focusing on carbonaceous buffer matrices incorporating Sn-carbon composites [23]. These carbon matrices meritoriously buffer the volume changes in Sn during the lithiation/delithiation process, and can effectively stabilize the structures of the electrodes, significantly enhancing the cycle performance [24,25,26]. Different types of carbon matrices have been employed in electrode materials, such as carbon nanotubes (CNTs) [27], carbon nanospheres, carbon nanofibers (CNFs) [28,29], graphene [30,31], graphite [32], etc., which have shown to be promising in terms of their enhanced electrochemical performance. Moreover, many studies have been conducted to control and manipulate the morphological structure of Sn, along with its carbonaceous matrices, to assist lithium ions during the charging/discharging progress. These morphological nanostructures are critical in preventing the agglomeration of electrode-active materials upon cycling. Liu et al. utilized the one-step dielectric barrier discharge oxygen plasma-assisted method to synthesize a nano-Sn@SnOx/C composite with a high reversible capacity retention of 500 mAhg^−1^ at 250 mAg^−1^ after 70 cycles [32]. Yunhua et al. fabricated nano-Sn/C composites employing the aerosol spray pyrolysis method, and the obtained nano-Sn/C composites exhibited a high reversible capacity of about 710 mAhg^−1^ at a higher current density of 200 mAg^−1^ without the capacity fading at up to 130 charge/discharge cycles [33]. In the literature, the Sn-based/C composite has exhibited favorable electrochemical performance with a stable cycle life for LIBs. Other research has been conducted on the direct incorporation of Sn-C composites as negative electrodes for lithium-ion batteries. Here, in this work, nanostructure Sn/C composite anodes were prepared, where glucose was used as the direct source of carbon by utilizing a facile hydrothermal method, along with post-heat treatment of LIBs. The as-prepared nanostructure Sn/C negative electrodes showed a high reversible capacity of 877 mAhg^−1^ at 0.1 Ag^−1^, with a high first cycle charge/discharge coulombic efficiency of 77%, and showed 668 mAhg^−1^ even at a relatively high current density of about 0.5 Ag^−1^ after 100 cycles. Based on the basic structural information of the Sn/C composite, its enhanced electrochemical performance and long lifespan are due to the fine structure and homogenous distribution of Sn in a carbon matrix. We think that Sn/C-based nanostructure electrodes may be effective for next-generation lithium-ion batteries.

## 2. Results

**Figure 1:**
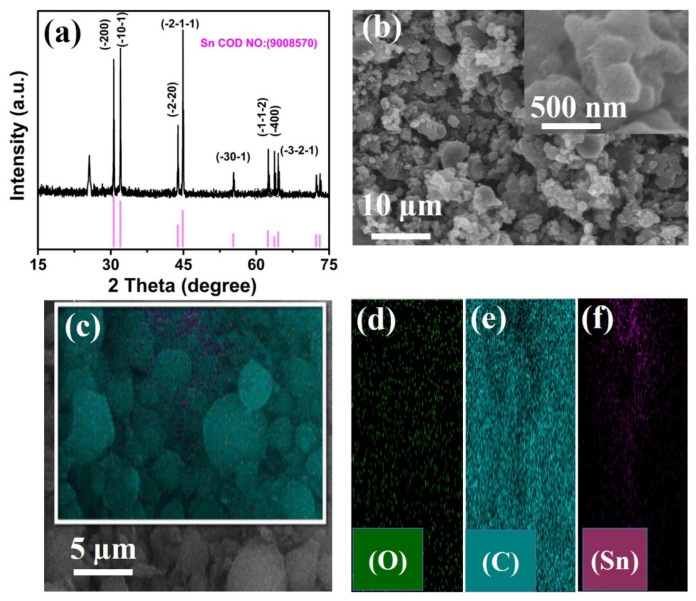
(**a**) XRD pattern of the Sn/C composite. (**b**) SEM images (low- and high-magnification images); (**c**) EDS and the corresponding elemental mapping (**d**–**f**) of the Sn/C composite.

By using the facile hydrothermal method, the Sn/C composite was prepared by following the experimental protocols along with a post heat treatment at 800 °C. The X-ray powder diffraction (XRD) method was employed to investigate and observe the crystalline nature of the as-prepared samples, as shown in Figure 1a. As shown in Figure 1a, all the diffraction peaks matched well with the single phase of tetragonal Sn (JCPDS No. 90-08570). A lower-intensity diffraction peak at around 25° could be seen in the XRD pattern, indicating the existence of carbon in the as-prepared composite. All the observed diffraction peaks for Sn had strong intensities, where a depressed intensity diffraction peak was observed for carbon in the synthesized Sn/C composite XRD pattern. There was no other crystal phase related to a tin oxide structure observed in the Sn/C composite [34]. The observed XRD pattern of the prepared sample was a single tin crystalline structure with high-intensity diffraction peaks, as presented in Figure 1a. Figure 1b shows the scanning electron microscope image of the as-synthesized Sn/C composite samples.

Figure 1b depicts a low-magnification image, along with an inset at high magnification to the right of the low-magnification image. The observed morphology of the sample contained irregular nanoparticles with different morphologies, which may directly reflect the heat treatment during the annealing process. Note, the irregular particles in size and inter-particles created short pathways in the composite for the penetration of electrolytes and Li-ions. Figure 1c–f show the elemental distribution analysis of the prepared Sn/C composite. It can be seen in the figure that tin particles were well-embedded into the amorphous carbon matrix. The dark spot in the figure represents carbon, while the light spot indicates tin nanoparticles. In addition, oxygen was observed in the light spot, which may have been caused by the exposure of materials in the air, as well as by the possible presence of amorphous tin suboxides (SnO_x_) in the composite. The presence of a carbon framework did not only inhibit the aggregation of Sn particles, but also alleviated volume expansion and improved the electronic conductivity of the entire electrode during lithiation/delithiation.

**Figure 2:**
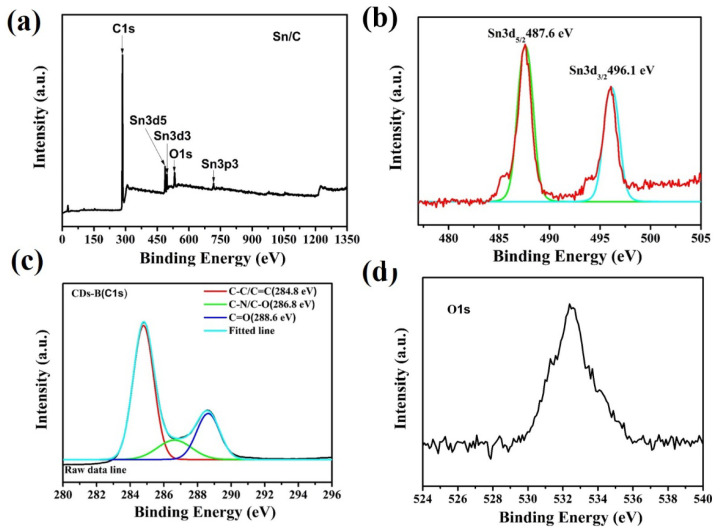
XPS spectra of Sn/C: (**a**) survey scan; (**b**) Sn3d spectra; (**c**) C1s spectra; (**d**) O1s spectra.

X-ray photo electron spectroscopy (XPS) was employed to further scrutinize the chemical composition and oxidation states of the as-prepared Sn/C composite, as shown in Figure 2a–d. In the low-resolution survey spectra depicted in Figure 2a, the typical peaks of carbon, tin, and oxygen can be identified, indicating the purity of the synthesized Sn/SnO_2_/C nanocomposite samples. As shown in Figure 2b, two strong characteristic peaks belonging to Sn3d_5/2_ and Sn3d_3/2_ of Sn/SnO_2_/C were observed at 487.2 and 496.6 eV, respectively [35]. These characteristic bands of Sn/SnO_2_/C confirm the oxidation of Sn into Sn^+4^ states [36]. Figure 2c represents the high-resolution spectra of Cs1 at 286.1 eV, which further confirm the existence of carbon in the composite sample, and can be deconvoluted into three individual component peaks. These component peaks contain C–C in aromatic rings at 284.8 eV, C–N at 286.8 eV, and C=O at 288.6 eV. The major peak of carbon in the spectra indicates the existence of C species in the Sn/C nanocomposite. Figure 2d shows the high-resolution spectra of Os1 with a maximum intensity of 533.6 eV, attributed to the C–O-bonding functional group, which further contributed to the reversibility of Li_2_O during the cycling process [37].

**Figure 3:**
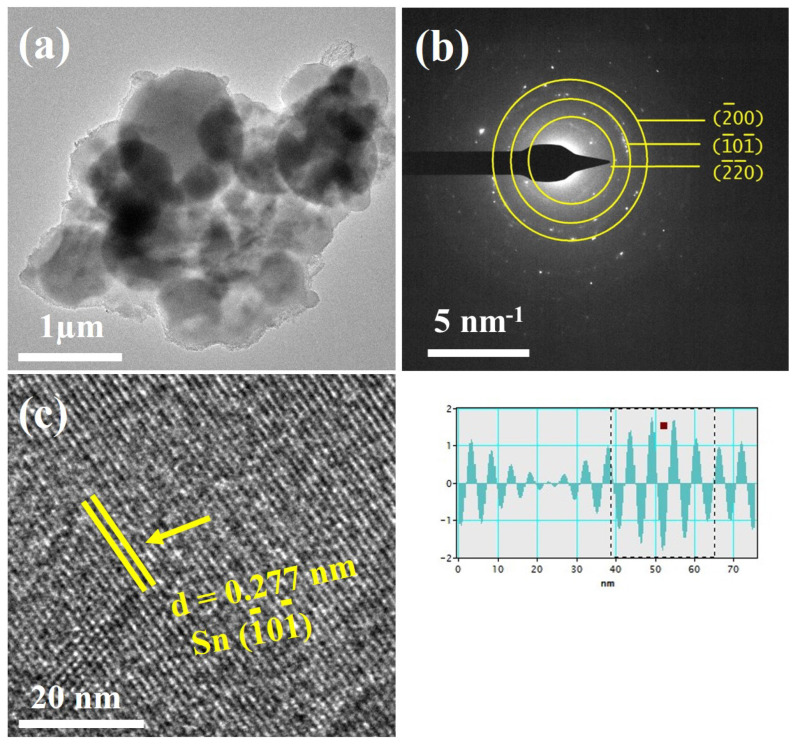
(**a**) TEM image of the Sn/C composite; (**b**) SAED pattern of the composite; (**c**) HRTEM-enlarged image of the composite.

To confirm the morphological details of the as-synthesized Sn/C composite, transmission electron microscopy, along with high-resolution TEM and selected area electron diffraction (SAED), were employed, and the results are shown in Figure 3. It can be seen in Figure 3a that tin nanoparticles were distributed in the carbon matrix, where the light spot represents the carbon, while the dark spot shows tin particles. It can be observed that all the particles were dispersed homogeneously inside the carbon matrix. Selected area electron diffraction (SAED) was employed to further investigate the single-crystalline nature of the tin particles embedded in the carbon framework (Figure 3b). All the diffraction spots confirmed the presence of tin particles in the carbon matrix, and the appearing diffraction rings from the Sn phase correspond to the (200), (101), and (220) crystal planes shown in Figure 3b. In addition, other diffraction spots were observed, which might have corresponded to other phases and may belong to tin oxide phases in the composites, but there was no evidence of this in the XRD pattern. Figure 3c illustrates the high-resolution TEM image of the composite, further showing the existence of the crystalline structure of Sn with its (101) plane. The identified d-spacing (0.277 nm) corresponding to the (101) plane perfectly matched the XRD results.

**Figure 4:**
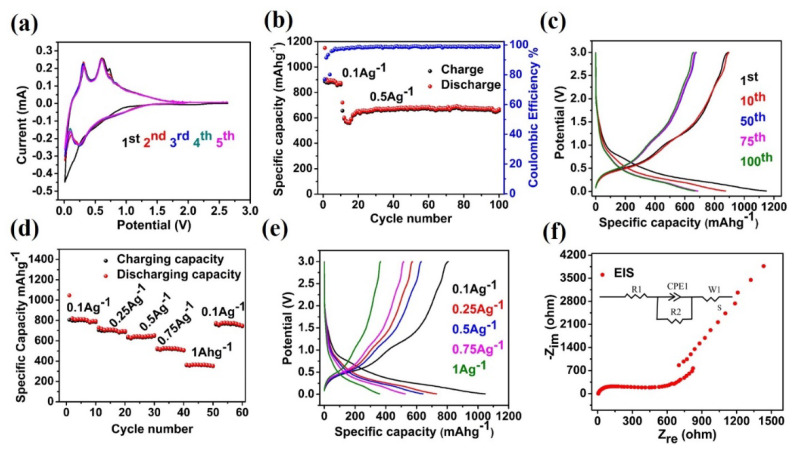
Electrochemical performance of the Sn/C composite electrode in LIBs. (**a**) Initial five CV curves scanned from 0.01 to 2.8 V at a rate of 0.01 mV s^−1^. (**b**) Cycle performance of the Sn/C electrode acquired at a current density of 100 mA g^−1^. (**c**) The corresponding discharge/charge profiles from selected cycles in (**b**). (**d**) Rate performance of Sn/C at various current densities from 0.1 to 1 A g^−1^. (**e**) The typical discharge/charge profiles at various current densities. (**f**) EIS of Sn/C.

The electrochemical performance of the as-prepared Sn/C composite used an active-working negative electrode in combination with Li as a reference and counter electrode in the half-coin cell. The investigated and corresponding cyclic voltammogram (CV), cycle performance, galvanostatic charge/discharge profiles of different cycles, rate performance, and electrochemical impedance spectroscopy (EIS) are thoroughly and systematically shown in Figure 4. Figure 4a illustrates the CV result of the Sn/C-negative electrode in the potential window 0.01–2.8 V (vs. Li^+^/Li) at a scanning rate of 0.1 mV s^−1^ for the initial five cycles for the LIBs. At the first cathodic cycle, there were no obvious reduction peaks observed contributing to the formation of an SEI layer on the surface of the electrode. From the second to the rest of the cycles, two reduction peaks were observed at 0.1 and 0.2 V, showing the presence of carbon in the composite. Additionally, all the observed peaks from the second cycle were distinct and well-overlapped, suggesting stable electrochemical reversibility during the subsequent cycles. Figure 4b,c show the representative cycle performance and selective galvanostatic charge/discharge performance of the Sn/C electrode for the LIBs from 1st to 100th cycles at two different current densities 0.1 and 0.5 Ag^−1^. The electrode showed a superior cycling performance and delivered a high reversible capacity up to 10 cycles, but then suddenly dropped to 559.17 mAhg^−1^ when the current densities switched to 1 Ag^−1^. After several cycles, the capacity reached a maximum range of about 677 mAhg^−1^, with a stable cycle performance for the remaining 90 cycles. The irreversible capacity of the initial charge/discharge cycle, shown in the corresponding galvanostatic charge in Figure 4c, was due to the SEI layer formation on the electrode surface of Sn/C, and was in agreement with the first cycle shown in the CV (Figure 4b). The charge/discharge capacities of the composite electrode were calculated based on the actual mass contained by the electrode of carbon and tin. The coulombic efficiency of the first charge/discharge was observed at about 77.01%. However, the corresponding coulombic efficiency reached its maximum range of about 99%, even at a high current density, and maintained capacity (677 mAhg^−1^) for the remaining cycles. In order to ensure the stable and superior electrochemical performance of the Sn/C composite, carbon additives were added during the preparation of the electrode. The stable cycle performance and repeatable charge/discharge profile showed that the Sn/C composite is a suitable negative electrode for Li-ion storage. Figure 4d,e show the rate performance of the Sn/C anodes for LIBs. A series of current densities of 0.1, 0.25, 0.5, 0.75, and 1 Ag^−1^ was employed to evaluate the rate capability of the Sn/C anode for LIBs. When applying the respective current densities on Sn/C composite electrode for LIBs, the electrode displayed different capacities of about 814, 684, 633, 507, and 348 mAhg^−1^, respectively.

When the current density was restored to 0.1 Ag^−1^, the average specific capacity of the Sn/C composite returned to 717 mAhg^−1^. Furthermore, the coulombic efficiencies of the Sn/C composite were as high as over 99%. The excellent rate performance could be primarily attributed to Li-ion transport upon the lithiation/delithiation of the homogenous distribution of tin particles in the carbon matrix. Figure 4f shows the electrochemical impedance spectra (EIS) result, which indicates the reason for the superior electrochemical performance of the Sn/C electrode during the cycle processes. EIS spectra usually contain a semicircle and straight line differentiating the charge transfer in high- and low-frequency ranges. The semicircle in the Nyquist plot could be attributed to the charge transfer in the high-frequency region, with a high resistance associated with the electrode and electrolyte interface, where the straight line represents the Li-ion diffusion in the low-frequency region inside the electrode bulk. The smaller semicircle indicates the low charge transfer of the Sn/C electrode. The EIS result in Figure 4f shows that Sn/C had the lowest resistance, demonstrating the structural compatibility of Sn and C by following the facile hydrothermal process to synthesize excellent composite electrodes with a tight interface for Li-ion storage. The excellent electrochemical performance of the Sn/C electrode for LIBs can be attributed to the following reasons: First, the composite of Sn and C created short pathways for Li-ion diffusion during lithiation/delithiation. Second, the existence of amorphous carbon buffered the volume expansion and contraction during lithiation/delithiation. Third, the formation of the SEI layer on the surface of the electrode and the decomposition of the electrolyte occurred during the first cycle. We conclude, based on the results obtained on electrochemical performance, that the Sn/C nanostructure composite used as an electrode can improve the mobility of the electrons in the presence of a carbon matrix and contribute to the capacity of LIBs.

## 3. Experimental Section

### 3.1. Preparation of Porous Sn/C Composite

A series of samples of Sn/C was prepared by using a facile hydrothermal method by obtaining the starting reagents: tin chloride dihydrate SnCl_2_.2H_2_O (99%, Aladdin Nanjing Chemical Reagent Co., LTO, Nanjing, China) and glucose C_6_H_12_O_6_ (99%, Aladdin Nanjing Chemical Reagent Co., LTO). All the reagents were of the analytical grade, and were used without any further purification. We mixed 0.02 mol (4.51 g) of tin chloride dehydrate SnC_l2_.2H_2_O as the tin source, and glucose C_6_H_12_O_6_ 0.02 mole (3.6 g) as the carbon source, in 50 mL of deionized water and stirred for 30 min with the help of a magnetic stirrer to produce a homogenous solution. The solution was then transferred to a 100 mL Teflon-lined autoclave and heated to 200 °C, and kept at this temperature for 24 h. After that, the solution was naturally cooled to room temperature, then centrifugated at 9000 r/min several times. In order to be purified, the product was washed with DI water and ethanol. The resultant samples were dried at 90 °C overnight, and then ground to powder. After that, the final materials were annealed at 800 °C for 4 h (5 °C min^−^^1^) under an argon atmosphere to get a Sn/C composite, and were naturally cooled to room temperature. These experimental steps were repeated in the same way for further authenticity and confirmation. The resultant materials were used further for basic structural characterization and investigating electrochemical performance.

### 3.2. Materials Characterization

The crystalline structures of the synthesized materials were examined by X-ray diffraction (XRD, Broker D8), with Cu Kα radiation (λ = 0.154 nm) in the range of 10–80 °C. Scanning electron microscopy (SEM FEI, Quanta 650) and TEM, along with selected area electron diffraction (SAED) and high-resolution transmission electron microscopy (HRTEM), were performed with a FEI Tecnai G2s-Twin instrument with an electron gun operating at 200 KV to further confirm the atomic distribution and size of the synthesized Sn/C materials. X-ray photoelectron spectroscopy (XPS, PHI-5000 VersaProbe Ulvac–Phi with an Al Kα radiation monochromator at 1486.6 eV) was performed to confirm the composition and oxidation state of the prepared Sn/C material.

### 3.3. Electrochemical Measurement

For the preparation of the Sn/C electrodes, 70% active material (Sn/C), 20% carbon black, and 10% PVDF as a binder were mixed for 40 min with the help of a pestle mortar. To produce a slurry, NMP solution was added into the mixed powder, then mixing continued until a homogenously thick liquid slurry was produced; then, it was pasted onto Cu foil. Electrodes were dried in a vacuum oven at 80 °C overnight. Before transferring to the glovebox, the electrodes were cut down into small, circular, 12 mm pieces and then pressed at 20 MPa. The average loading mass of the active-material Sn/C composite was 1.4 mg. Lithium foil was used both as the reference and counter electrode, a Celgard 2400 membrane was used as a separator, and 1 M LiPF_6_ in ethylene carbonate (EC) and diethyl carbonate (DEC) with a volume ratio of EC:DEC = 1:1 were used as the electrolyte. CR-2032 half-coin cells were used for assembling cells inside an Ar-filled glovebox. The specific capacity of the LIBs was tested on a Land automatic battery tester (LAND-CT2001A, Wuhan, China). Cyclic voltammetry (CV) and electrochemical impedance spectroscopy (EIS) were performed on an electrochemical workstation (CHI660D, Shanghai, China).

## 4. Conclusions

The Sn/C nanocomposite was synthesized via a highly productive facile hydrothermal method. The synthesized Sn/C nanocomposite was used as a negative electrode material for lithium-ion batteries. This nanocomposite material exhibited outstanding electrochemical performance, including stable cyclability and rate capability when tested at a relatively high current density of up to 1 Ag^−1^, which revealed an excellent Li storage capacity of about 877 mAhg^−1^ at 0.1 Ag^−1^. The presence of carbon in the composite electrode does not only enhance and stabilize the electrochemical performance in terms of high stability with high coulombic efficiency, but also increases the electronic conductivity of the entire electrode and buffers the volume expansion during the lithiation/delithiation process. Furthermore, the basic structural and morphological characterizations, observed with XRD, SEM, and TEM, confirmed the uniform and homogenous dispersion of single-phase Sn nanoparticles in the conductive carbon matrix. Considering the low cost and eco-friendly preparation via the facile hydrothermal method, and its attractive electrochemical performance, the Sn/C nanocomposite prepared in this work can be used as an effective negative electrode for next-generation LIBs.

## Data Availability

Data sharing is not applicable for this article.
